# Key virulence genes associated with *Streptococcus mutans* biofilm formation: a systematic review

**DOI:** 10.3389/froh.2025.1654428

**Published:** 2025-08-26

**Authors:** Dinda Kurnia Fitri, Nozimjon Tuygunov, Wan Himratul Aznita Wan Harun, Isty Adhitya Purwasena, Arief Cahyanto, Myrna Nurlatifah Zakaria

**Affiliations:** ^1^ Department of Restorative Dentistry, Faculty of Dentistry, Universiti Malaya, Kuala Lumpur, Malaysia; ^2^ Department of Oral Biology, Faculty of Dentistry, University of Jenderal Achmad Yani, Cimahi, Indonesia; ^3^ Faculty of Dentistry, Kimyo International University in Tashkent, Tashkent, Uzbekistan; ^4^ Department of Oral & Craniofacial Sciences, Faculty of Dentistry, Universiti Malaya, Kuala Lumpur, Malaysia; ^5^ Department of Microbiology, School of Life Sciences and Technology, Institut Teknologi Bandung, Bandung, Indonesia; ^6^ Department of Clinical Sciences, College of Dentistry, Ajman University, Ajman, United Arab Emirates; ^7^ Centre of Medical and Bio-allied Health Sciences Research, Ajman University, Ajman, United Arab Emirates

**Keywords:** *Streptococcus mutans*, virulence genes, biofilm formation, dental caries, dental plaque, glucosyltransferase

## Abstract

**Introduction:**

*Streptococcus mutans* is central to plaque-induced oral diseases due to its robust biofilm-forming ability. Understanding the genetic and regulatory basis of this process is critical for developing targeted anti-virulence strategies that preserve the balance of the oral microbiome. This systematic review aims to gather and evaluate existing evidence on the virulence genes associated with *Streptococcus mutans* biofilm formation.

**Methods:**

A comprehensive search of PubMed, Scopus, and Web of Science was conducted in accordance with PRISMA guidelines. Studies investigating the genetic and regulatory mechanisms of biofilm formation, as well as the effects of experimental treatments, were included, and the risk of bias was assessed using the QUIN tool.

**Results:**

Key virulence genes were identified, including glucosyltransferases *(gtfB, gtfC, gtfD)*, glucan-binding proteins *(gbpB, gbpC)*, and two-component systems *(vicRK, liaSR)*. These genes contribute to adhesion, extracellular polysaccharide synthesis, and environmental adaptation, processes critical for biofilm development. Various anti-virulence strategies, such as quorum sensing inhibitors and gene-targeted compounds, show promise in controlling biofilm formation without compromising bacterial viability, thereby preserving the homeostasis of the normal oral flora, which is essential for maintaining overall oral health.

**Conclusion:**

While key virulence genes have been well characterized, further research is needed to clarify how their regulation is influenced by environmental conditions. Insights from this review may support the development of novel therapeutic approaches that reduce *Streptococcus mutans* pathogenicity while maintaining oral microbial balance.

**Systematic Review Registration:**

https://www.crd.york.ac.uk/PROSPERO/view/CRD42024577977, PROSPERO CRD42024577977.

## 1 Introduction

Dental caries is recognized as one of the most widespread global oral health issues, affecting an estimated 2 billion people according to the World Health Organization (WHO) (1). Oral health disparities remain significant, particularly in low- and middle-income countries (2). *Streptococcus mutans* is among the pathogens of concern for WHO due to its increasing multidrug resistance (MDR) and its key role in oral infections (3). Addressing the virulence mechanisms of *S. mutans*, particularly its biofilm formation capabilities, is thus critical not only for individual patient health but also for public health initiatives aimed at improving global oral health outcomes (4). Targeting biofilm formation through advanced strategies, including the integration of Artificial Intelligence driven screening for biofilm inhibitors, fluoride-based interventions, or bioactive dental materials, represents promising avenues for future preventive strategies in dental caries management at the global scale (5–8).

Dental plaque is a biofilm that forms on the tooth surface through interactions between oral bacteria, their metabolic byproducts, saliva and diet (9, 10). The structural organization of a biofilm provides several advantages to bacteria such as protection from antimicrobial agents and the host immune system, enhanced co-aggregation, and specific interaction preferences. These protective mechanisms make the biofilm a challenging target for therapeutic interventions (11, 12).

Biofilm formation generally progresses through four distinct stages: 1. the adhesion of bacteria to a surface, 2. the development of microcolonies, 3. the maturation of the biofilm structure, and 4. detachment or dispersal, which facilitates bacterial colonization of new environments (13). The initial adhesion of bacterial cells is a critical stage in biofilm formation. After adhesion, bacteria may follow one of two pathways influenced by environmental conditions: they may remain attached and progress to biofilm development, or they may revert to a planktonic state (11). Biofilms are highly dynamic ecosystems, with cells continuously detaching from the main structure either actively or passively. These dispersed cells can colonize new surfaces and form fresh bacterial colonies. Bacteria within the biofilm, known as sessile bacteria, typically exist in a stationary or dormant growth phase and exhibit phenotypic traits distinct from those of planktonic bacteria (13, 14). Bacteria within biofilms exhibit exceptional resistance to environmental stresses, particularly antibiotics. This resistance makes biofilms a significant public health concern, as they are responsible for 60%–80% of human microbial infections (12, 14).

*Streptococcus mutans* plays a central role in dental plaque formation and is closely associated with the development of oral diseases such as dental (15). The plaque-forming and cariogenic potential of *S. mutans* is widely recognized to stem from three key attributes: 1. its ability to synthesize large amounts of extracellular glucan polymers from sucrose, which facilitate permanent colonization of hard surfaces and the formation of the extracellular polymeric matrix *in situ*, 2. its capacity to transport and metabolize a broad range of carbohydrates into organic acids (acidogenicity), and 3. its ability to survive under environmental stress, particularly in low pH conditions (aciduricity) (15, 16). Key contributors to its biofilm-forming capability include glucosyltransferases (gtfB, gtfC, gtfD), which synthesize extracellular glucans that promote adhesion and structural integrity of the plaque biofilm, as well as regulatory systems like vicRK, which influence biofilm maturation and stress response.

Recent studies have explored the potential of anti-virulence agents, such as shikimic acid and betulin, to downregulate key virulence genes involved in biofilm formation without affecting bacterial viability (17, 18). These findings underscore the need for a comprehensive understanding of the specific virulence genes involved in biofilm formation and their regulatory mechanisms in *S. mutans* biofilm formation. This systematic review aims to gather and evaluate current evidence on the virulence genes associated with *S. mutans* biofilm formation. By examining their regulatory mechanisms and functional roles, this review seeks to provide insight into potential therapeutic targets that could reduce biofilm-associated pathogenicity while maintaining the ecological balance of the oral microbiome.

## 2 Methods

### 2.1 Research strategy

This systematic review was registered in PROSPERO with the registration number CRD42024577977 and was carried out following the guidelines of the Preferred Reporting Items for Systematic Reviews and Meta-Analyses (PRISMA). Article selection and data extraction were performed by two independent reviewers (D.K.F and N.T) comprehensively, using three electronic databases: Scopus, Web of Science, and PubMed MEDLINE. Free-text and MeSH terms were applied to the search, using Boolean operators (OR, AND) to optimize term combinations, as outlined in Table 1. Manual searches were additionally performed to ensure comprehensive coverage of relevant literature. All reviewers independently screened the titles and abstracts of the search results, with any discrepancies discussed and resolved collaboratively.

**Table 1:** Keywords used in searching for the appropriate article.

Keywords
(“Streptococcus mutans”) AND (“Virulence Genes” OR “Pathogenicity”) AND (“Biofilm Formation” OR “Bacterial Adhesion”) AND (‘'Dental Caries’‘ OR “Dental Plaque") AND (“*in vitro*” OR “quasi-experimental study”)

### 2.2 Eligibility criteria

The eligibility criteria for each study type were defined using the PICOS framework, which considers Population/Problem, Intervention, Comparison, Outcome, and Study design. This structured approach, outlined in Table 2, was designed to ensure the inclusion of studies that are both reliable and relevant to the objectives of the review.

**Table 2:** PICOS criteria for inclusion of studies.

Component of PICOS Question	Inclusion	Exclusion
Problem/Population	Studies that investigate the regulation of genes of *S. mutans* associated with biofilm formation	Studies focusing on other bacterial species or not specifically investigating the regulation of *S. mutans* virulence genes associated with biofilm formation.
Intervention	Studies investigating the role of *S. mutans* virulence factors in biofilm formation, focusing on experimental treatments or strategies to regulate the expression of biofilm-associated genes	Studies that do not investigate gene expression or are unrelated to the role of *S. mutans* virulence factors in biofilm formation, including those lacking experimental treatments or strategies aimed at regulating biofilm-associated gene expression
Comparison	Comparisons between intervention (e.g., treatments targeting *S. mutans*) with control groups (e.g., placebo or no treatment) in relation to biofilm formation and the expression of biofilm-related genes.	Studies comparing interventions unrelated to *S. mutans* biofilm formation or virulence factors, or those lacking a comparative component (e.g., no control or reference group).
Outcome	*In vitro* studies related to *S. mutans* virulence factors in biofilm formation, focusing on changes in biofilm characteristics through quantitative or qualitative assessments, and/or evaluating the expression levels of key biofilm-associated virulence genes.	*In vivo* studies, or studies lacking quantitative or qualitative assessment of biofilm formation and/or virulence gene expression in *S. mutans*.
Study Design	Experimental and quasi-experimental studies, articles published not more than ten years, papers in Q1 and Q2 journals.	Reviews, systematic reviews, book chapters, conferences, non-English article, articles published more than ten years, and papers published in Q3 and Q4 journals.

### 2.3 Data extraction

The primary and secondary reviewers reached a consensus to extract the necessary data from reputable scientific databases, such as Scopus, Web of Science, and PubMed MEDLINE. Before data extraction, the keywords for the search were clarified and approved by the third and fourth authors. Reviewer 1 collects the data in.csv format and imports it into an Excel file to create a table. The table includes seven columns: authors, title, publication year, source title, abstract, link (or DOI), and comments. In parallel, Reviewer 2 independently performs a similar task, adhering to the same inclusion/exclusion criteria. This process ensures the accurate selection of papers and minimizes the risk of errors.

### 2.4 Risk of bias assessment

Two reviewers (D.K.F and N.T) used the Quality Assessment Tool for *In vitro* Studies (QUIN tool) to assess the risk of bias in the selected studies. The QUIN tool provides a standardized approach to evaluating the risk of bias in *in vitro* studies included in systematic reviews and meta-analyses. It has been tested for content validity and includes 12 criteria. Each criterion is given a score: 2 points for adequately specified, 1 point for inadequately specified, 0 points for not specified, and N/A (not applicable) for criteria excluded from the calculation. The scores for all 12 criteria are then added up to give a total score for the study. Based on this total score, studies are categorized into three risk levels: high (<50%), medium (50%–70%), or low (>70%) risk. The categorization is determined by the formula: Final score = (Total score × 100)/(2 × number of applicable criteria) (19).

## 3 Results

### 3.1 Search result

**Figure 1:**
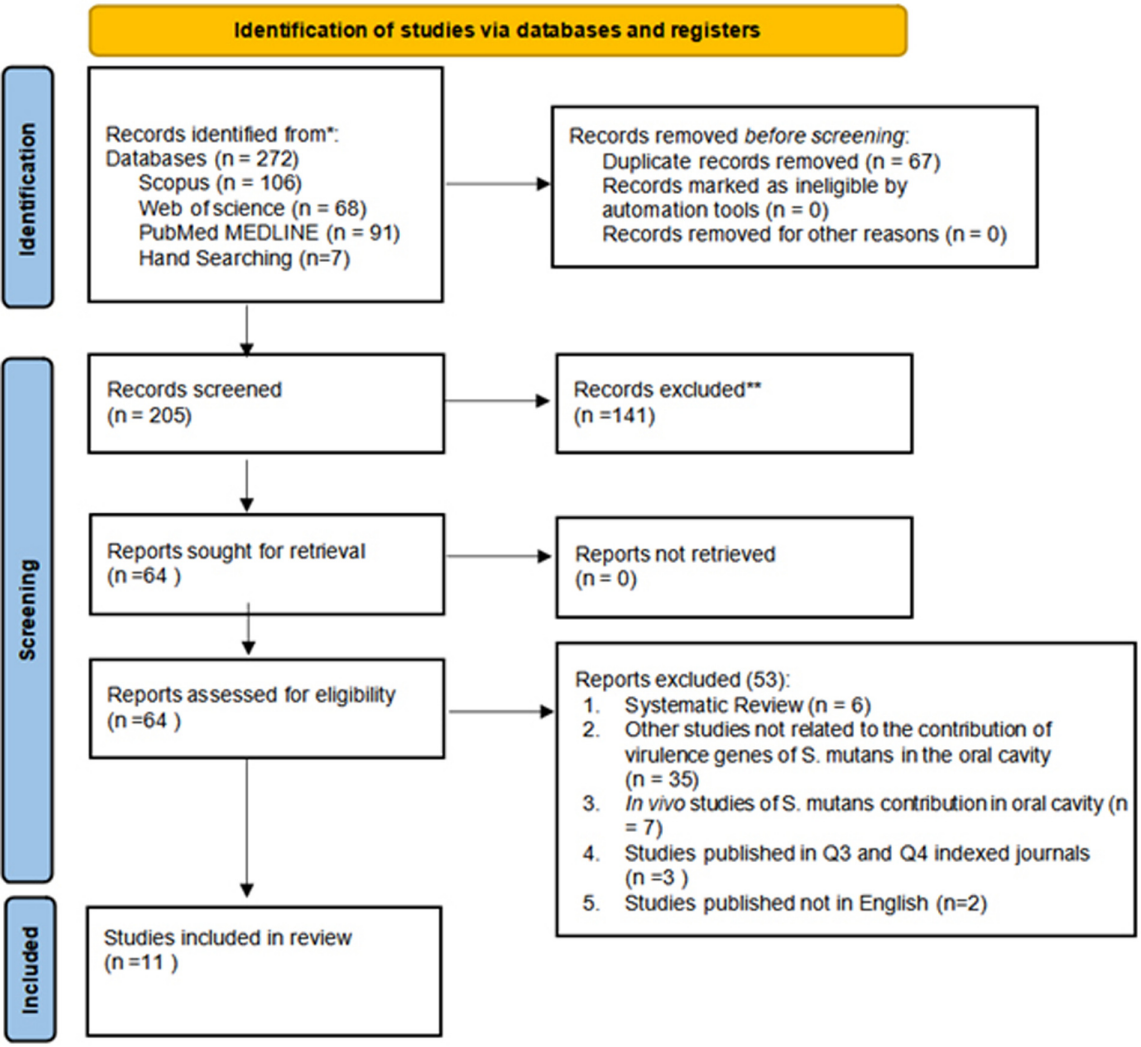
PRISMA 2020 flow diagram illustrating the study selection process. Adapted from Page MJ, Moher D, Bossuyt PM, Boutron I, Hoffmann TC, Mulrow CD, et al. PRISMA 2020 Explanation and elaboration: updated guidance and exemplars for reporting systematic reviews (20). Licensed under CC BY 4.0.

The study selection process adhered to a predefined framework of inclusion and exclusion criteria based on the PICOS scheme, targeting research on virulence genes involved in biofilm formation by *Streptococcus mutans*. An initial search identified 272 studies through keyword screening from three databases: Scopus (*n* = 106), Web of Science (*n* = 68), Pubmed/Medline (*n* = 91) and seven articles were selected from hand searching. Following a detailed evaluation, 11 *in vitro* studies met the eligibility criteria and published in Q1 and Q2 indexed journals within the last decade (2014–2024). The selection process for this review is presented in Figure 1 (20).

### 3.2 Risk of bias and quality assessment

**Figure 2:**
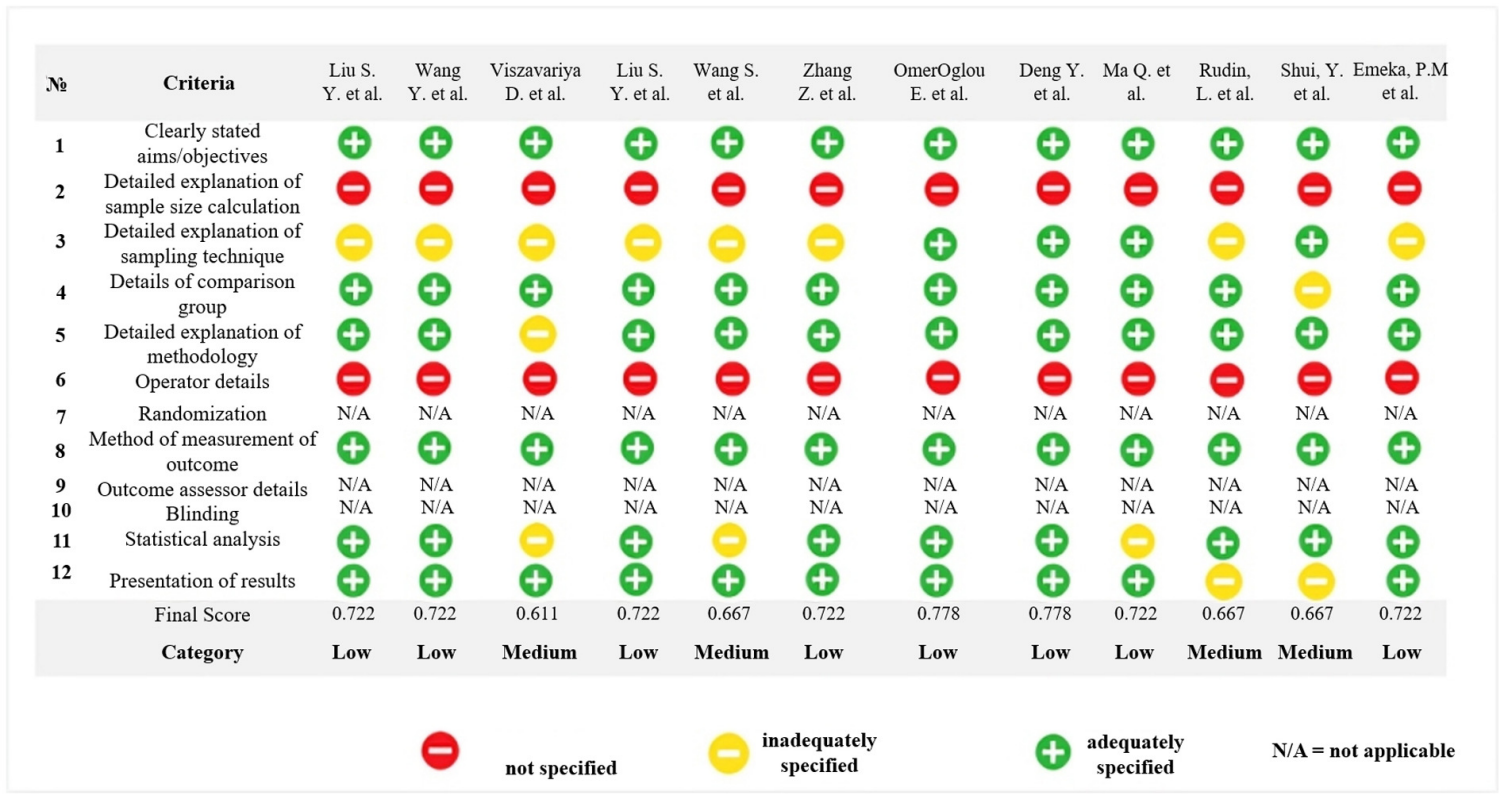
Methodological evaluation of *in vitro* assays according to the quality assessment tool for *in vitro* studies (QUIN).

The risk of bias (RoB) assessment was independently conducted by two reviewers (D.K.F and N.T) using the QUIN tool for *in vitro* studies (19). Six studies were classified as having low risk of bias (21–26), while the remaining four studies were classified as having medium risk of bias as presented in Figure 2 (17, 18, 27, 28). As a result, all studies included in this review surpassed 50% of the evaluated criteria. However, the assessment using the QUIN tool highlighted a consistent limitation across all selected studies: none provided details regarding the method used to calculate the sample size or operator details. Figure 2 illustrates the results of the methodological evaluation of *in vitro* assays according to the Quality Assessment Tool for *In vitro* Studies (QUIN).

All eleven studies examined the gene expression of *S. mutans* related to biofilm formation, quorum sensing, adherence, EPS regulation, and virulence using RT-qPCR method with various tested substances. Four of these studies used broth supplemented with different sucrose concentrations in the biofilm formation process (17, 18, 21, 28). Most of the studies reported downregulation of the targeted genes when treated with the respective test materials compared to the untreated group as the negative control. The virulence genes, experimental methods, and key findings from the included studies on *Streptococcus mutans* biofilm formation are summarized in Table 3. Table 4 presents the corresponding primer sequences for the identified genes.

**Table 3:** Overview of included studies on virulence genes associated with *Streptococcus mutans* biofilm formation.

Study ID	Treatment	Strain	Medium	Gene	Methodology	Result
(21)	Sucrose	*S. mutans* UA159	BHI broth with 1% sucrose or 5% sucrose	GtfB	*S. mutans* UA159 was incubated in BHI broth with 1% or 5% sucrose under anaerobic conditions, and growth was monitored by absorbance at 600 nm and pH changes. Total RNA was extracted at the late exponential/early stationary phase using the miRNeasy Mini Kit.	Six of the 22 differentially expressed sRNAs were validated, with the target gene gtfB showing higher expression in 1% sucrose compared to 5% sucrose.
				GtfC		
				16s rRNA		
(64)	AMP GH12	*S. mutans* UA159	BHI broth, Tryptone-yeast extract medium	ldh	qRT-PCR of *S. mutans* grown with sub-MIC levels of GH12 was studied, with untreated bacteria as the control. RNA was extracted and purified, and gene expression was normalized to 16S rRNA using the 2−*ΔΔ*Ct method.	ldh, gtfBCD, vicR, liaR, and comDE genes were significantly downregulated.
				atpD		
				gtfBCD		
				vicR		
				liaR		
				comDE		
				16s RNA		
(17)	Betulin	*S. mutans* UA159	Tryptose agar plates, Todd Hewitt Broth (THB) supplemented with 0.5% yeast extract and 1% sucrose	vicR	qRT-PCR of *S.* *mutans*	RT-qPCR revealed a marked downregulation of cariogenic gene expression.
				gbpB	grown in the presence and absence of betulin was performed, with RNA isolated using the guanidine thiocyanate/phenol extraction method. cDNA was synthesized using the High Capacity cDNA Reverse Transcription Kit, and gene expression was normalized to 16S rRNA using the 2−*ΔΔ*Ct method.	
				gtfBCD		
				smu0630		
				comDE		
				16S rRNA		
(23)	Mutating proline and histidine	*S. mutans* UA159	THYE media	gtfBCD	qRT-PCR of mid-log phase *S. mutans* cells was performed, with RNA isolated using Trizol and the Fast Prep system. cDNA synthesis was done using a first-strand cDNA synthesis kit, and gene expression was normalized to 16S rRNA.	The VicK P222A mutant reduced phosphatase activity, resulting in the downregulation of key genes, including gtfBC and SpaP.
				ftf		
				vicRK		
				SpaP		
				16S rRNA		
(18)	Shikimic Acid (SA)	*S. mutans UA159*	Brain Heart Infusion (BHI) broth, BHIS (BHI broth containing 1% sucrose)	gtfBCD	qRT-PCR of *S. mutans* treated with different concentrations of SA (1.6, 0.8, and 0.4 mg/ml) was studied, with RNA extracted using TRIzol and cDNA synthesized using the PrimeScript RT reagent kit. Gene expression was normalized to 16S rRNA and analyzed using the 2−*ΔΔ*Ct method.	Expression levels of gtf genes in *S. mutans* treated with SA were downregulated compared to the control group.
				16SrRNA		
(24)	Rhodiola rosea extract (RE)	*S. mutans* UA159	Brain Heart Infusion (BHI) broth	gtfBCD	qRT-PCR of *S. mutans* treated with RE at concentrations of 0.50, 0.25, and 0.12 *μ*g/*μ*l was performed to assess the expression of virulence gtf genes. RNA was extracted using TRIzol, and gene expression was normalized to 16S rRNA using the 2−*ΔΔ*Ct method.	The relative expression levels of gtf genes in the RE-treated groups were significantly decreased, indicating downregulation of gtf gene expression, with slight decreases in the expression of comDE also observed.
				comDE		
				16S rRNA		
(25)	Microbiota-derived postbiotic mediators (PMs)	*S. mutans* ATCC 25,175	Brain Heart Infusion (BHI) broth	gtfC	qRT-PCR of *S. mutans* cells treated with or without PMs was performed, with RNA extracted using PureZOL™ and cDNA synthesized using the iScript cDNA Reverse Transcription Kit. Gene expression was normalized to 16S rRNA and quantified using the 2−*ΔΔ*Ct method.	Postbiotic mediators (PMs) from *Lactiplantibacillus* strains, particularly *L. plantarum* EIR/IF-1, effectively downregulated key cariogenic genes without inhibiting bacterial growth.
				comAX		
				16S rRNA		
(28)	Natural flavone luteolin	*S. mutans* ATCC 25,175, *S. sanguinis* ATCC 10,556, *S. gordonii* ATCC 10,558, *S. mitis* ATCC 49,456, *S. oralis* ATCC 35,037	Brain Heart Infusion (BHI) broth	spaP	qRT-PCR of *S. mutans* biofilms treated with 25 μg/ml luteolin for 24 h was performed, with RNA extracted using TRIzol and cDNA synthesized using the GoScript™ Reverse Transcriptase system. Gene expression was normalized to 16S rRNA, and mRNA levels were quantified using the FastStart Universal SYBR Green Master and analyzed with the 2−*ΔΔ*Ct method.	Luteolin downregulated key virulence genes (gbpC, spaP, gtfBCD, ftf), which led to reduced production of surface adhesins and extracellular polysaccharides (EPS), and interfered with glucosyltransferases (Gtfs), decreasing the synthesis of water-insoluble glucans
				gbpC		
				gtfBCD		
				ftf		
				16S rRNA		
(28)	Sodium New Houttuyfonate (SNH)	*S. mutans* UA159	Brain Heart Infusion (BHI) broth, BHIS (BHI broth containing 1% [wt/vol] sucrose)	gtfBCD	qRT-PCR of *S. mutans* cultured in BHI broth supplemented with SNH (100 μg/ml) for 24 h was performed, with RNA extracted using RNAiso Plus and cDNA synthesized using the PrimeScript RT Reagent Kit. Gene expression was normalized to 16S rDNA, and relative mRNA levels were quantified using the 2−*ΔΔ*Ct method.	SNH downregulated the expression of gtfBCD and comDE systems and demonstrated synergistic effects with chlorhexidine (CHX).
				comDE		
				16S rRNA		
(26)	Extracts of Mangifera indica	*S. mutans* MTCC 890	Brain heart infusion (BHI) broth	gtfBCD	qRT-PCR of *S. mutans* biofilms treated with 0, 500, and 1,000 µM mangiferin was performed, with RNA extracted using TRIzol and cDNA synthesized using the Takara cDNA synthesis kit. Gene expression was normalized to 16S rRNA and quantified using the *ΔΔ*Ct method, with PCR carried out on a Qiagen Rotor-Gene Q thermal cycling system.	Mangiferin significantly downregulated key virulence genes (gtfBCD, gbpB, and comDE), all of which are crucial for biofilm formation and bacterial adherence.
				gbpB		
				comDE		
				16S rRNA		
(65)	farnesol and/or myricetin	*S. mutans* UA159	ultra-filtered tryptone-yeast extract (UFTYE) broth containing 1% sucrose	gtfBCD	Quantitative reverse transcription-PCR (qRT-PCR) was used to measure the expression of *Streptococcus mutans* target genes (gtfB, gtfC, gtfD, atpD). Biofilms were formed on sHA discs suspended in 24-well plates for 19 h at 37°C with 5% CO₂. Treatments were applied to the biofilms at 20 h using NPC and drug solutions, followed by an additional 4-hour incubation. RNA was extracted and purified, and cDNA synthesis was performed using the Bio-Rad iScript cDNA synthesis kit. Relative gene expression was quantified using Bio-Rad iTaq SYBR green Supermix and normalized to 16S rRNA.	The NP25/10 nanoparticles co-loaded with 4.5 mM farnesol and 1.0 mM myricetin reduced atpD gene expression by 43%, while gtfB, gtfC, and gtfD expression showed a non-significant decreasing trend. Myricetin, alone or with farnesol (without nanoparticles), showed the highest GtfB activity inhibition (37%–71%). In contrast, NP25/10 alone or with farnesol reduced GtfB activity by ∼20%, but the co-loaded formulation with a high myricetin concentration showed no significant effect.
				atpD		
				16S rRNA		

**Table 4:** Virulence genes associated with *Streptococcus mutans* biofilm formation.

Gene	Primer sequence	Role	Reference
GtfB	(F) GATCAAGATGTTCGCGTTGC	Synthesize insoluble glucans, facilitate adhesion to tooth surfaces.	(17, 18, 21, 23, 24, 26, 28, 64, 65)
	(R) ACACATACTGCGGTGCCATT		
	(F) CACTATCGGCGGTTACGAAT		
	(R) CAATTTGGAGCAAGTCAGCA		
	(F) AAAGCAACGGATACAGGGGA		
	(R) CTCTGTCATTGGTGTAGCGC		
	(F) ACACTTTCGGGTGGCTTG		
	(R) GCTTAGATGTCACTTCGGTTG		
	(F) AGCCGAAAGTTGGTATCGTCC		
	(R) TGACGCTGTGTTTCTTGGCTC		
	(F) CACTATCGGCGGTTACGAAT		
	(R) CAATTTGGAGCAAGTCAGCA		
	(F) AGCAATGCAGCCAATCTACAAAT		
	(R) ACGAACTTTGCCGTTATTGTCA		
	(F) AGCAATGCAGCCAATCTACAAAT		
	(R) ACGAACTTTGCCGTTATTGTCA		
GtfC	(F) GATCAAGAAGCGGCTGGTTT	Synthesize both soluble and insoluble glucans, facilitate adhesion to tooth surfaces.	(17, 18, 21, 23–25, 28, 64)
	(R) ACATGACGCGTGAATCAAGG		
	(F) GATGCTGCAAACTTCGAACA		
	(R) TATTGACGCTGCGTTTCTTG		
	(F) GGTTTAACGTCAAAATTAGCTGTATTAGC		
	(R) CTCAACCAACCGCCACTGTT		
	(F) CCAAAATGGTATTATGGCTGTCG		
	(R) TGAGTCTCTATCAAAGTAACGCAG		
	(F) GCCAAGTATGGGGGAGCTTT		
	(R) CATCGGAACCCCTGTGGAAA		
	(F) GTGCGCTACACCAATGACAGAG		
	(R) GCCTACTGGAACCCAAACACCTA		
	(F) GATGCTGCAAACTTCGAACA		
	(R) TATTGACGCTGCGTTTCTTG		
	(F) TTCCGTCCCTTATTGATGACATG		
	(R) AATTGAAGCGGACTGGTTGCT		
	(F) CTCAACCAACCGCCACTGTT		
	(R) GGTTTAACGTCAAAATTAGCTGTATTAGC		
GtfD	(F) TTGACGGTGTTCGTGTTGAT	Synthesize soluble glucans, source of nutrient for *S. mutans* and other bacteria.	(17, 18, 23, 24, 26, 28, 64, 65)
	(R) AAAGCGATAGGCGCAGTTTA		
	(F) TTAAATATGACTGTTGCTAGCTTATTG		
	(R) GGTTTCCATAAAATATCCTCCTTTATC		
	(F) GAAGTATGGCGGTGCTTTCC		
	(R) ATAACCAACACCACGGCCTA		
	(F) TTGACGGTGTTCGTGTTGAT		
	(R) AAAGCGATAGGCGCAGTTTA		
	(F) CACAGGCAAAAGCTAAATTAACA		
	(R)GAATGGCCGCTAAGTCAACAG		
	(F) TGGCACCGCAATATGTCTCTTC		
	(R) CAATCCGCAATAACCTGAATACCG		
Ldh	(F) AAAAACCAGGCGAAACTCGC	Contributes to the acidic environment within the biofilm, promote the growth of aciduric bacteria.	(64)
	(R) CTGAACGCGCATCAACATCA		
atpD	(F) TGTTGATGGTCTGGGTGAAA	Providing energy essential for maintaining metabolic activity and nutrient exchange in the mature biofilm	(65)
	(R) TTTGACGGTCTCCGATAACC		
VicR	(F) CGTGTAAAAGCGCATCTTCG	Part of two-component system, influence acid tolerance, involved in the stress response.	(17, 23, 64)
	(R) AATGTTCACGCGTCATCACC		
	(F) TGACACGATTACAGCCTTTGATG		
	(R) CGTCTAGTTCTGGTAACATTAAGTCCAATA		
	(F) CGCAGTGGCTGAGGAAAATG		
	(R) ACCTGTGTGTGTCGCTAAGTGATG		
VicK	(F) CACTTTACGCATTCGTTTTGCC	Part of two-component system, influence acid tolerance, involved in the stress response.	(23)
	(R) CGTTCTTCTTTTTCCTGTTCGGTC		
liaR	(F) CATGAAGATTTAACAGCGCG	Involved in the stress reponse, enhancing survival during the transition to biofilm development.	(23)
	(R) CGTCCTGTGGCACTAAATGA		
comA	(F) ACGAGCCTAACAAGGGGATT	Quorum sensing-an ABC transporter	(25)
	(R) CCCTGAGGCATTTGTTCAAT		
comD	(F) TTCCTGCAAACTCGATCATATAGG	A quorum-sensing system that coordinates gene expression in biofilm communities, regulating the production of extracellular enzymes and stress responses.	(17, 23, 24, 26, 28)
	(R) TGCCAGTTCTGACTTGTTTAGGC		
	(F) TTCCTGCAAACTCGATCATATAGG		
	(R) TGCCAGTTCTGACTTGTTTAGGC		
comE	(F) TTCCTCTGATTGACCATTCTTCTG	A quorum-sensing system that coordinates gene expression in biofilm communities, regulating the production of extracellular enzymes and stress responses.	(17, 24, 26, 28, 64)
	(R) GAGTTTATGCCCCTCACTTTTCAG		
	(F) TTCCTCTGATTGACCATTCTTCTG		
	(R) GAGTTTATGCCCCTCACTTTTCAG		
comX	(F) CTGTTTGTCAAGTGGCGGTA	Quorum sensing-an alternative sigma subunit of RNA polymerase	(25)
	(R) GCATACTTTGCCTTCCCAAA		
gbpB	(F) ATGGCGGTTATGGACACGTT	Helps maintain its cell-associated in the lack of a cell-wall anchor	(17, 26)
	(R) TTTGGCCACCTTGAACACCT		
	(F) ATGGCGGTTATGGACACGTT		
	(R) TTTGGCCACCTTGAACACCT		
gbpC	(F) GGCGATCATGTGGAAAAAGT	Develop dextrin-dependent aggregation (DDAG) *in vitro* under stressful conditions	(28)
	(R) ATAATAAGCCGTCGCAGCAC		
Ftf	(F) ATTGGCGAACGGCGACTTACTC	Synthesizes fructans, acting as energy reservoirs and enhancing biofilm robustness.	(23, 28)
	(R) CCTGCGACTTCATTACGATTGGTC		
	(F) AAATATGAAGGCGGCTACAAC		
	(R) AAATATGAAGGCGGCTACAAC		
SpaP	(F) GACTTTGGTAATGGTTATGCATC	Mediates bacterial binding to salivary glycoproteins and enamel	(23, 28)
	(R) TTTGTATCAGCCGGATCAAGT		
	(F) GCTCATAAAGCCGAGGTTG		
	(R) CAGCCTGATAAGCAGCAAG		
smu0630	(F) GTTAGTTCTGGTTTTGACCGCAAT	Contributes to the dense, cohesive biofilm matrix in the mature phase.	(17)
	(R) CCCTCAACAACAACATCAAAGGT		
16s rRNA	(F) AGCGTTGTCCGGATTTATTG	Houskeeping gene	(17, 18, 23, 24, 26, 28, 64, 65)
	(R) CTACGCATTTCACCGCTACA		
	(F) ACTCCTACGGGAGGCAGCAG		
	(R) ATTACCGCGGCTGCTGG		
	(F) CTGACTTGAGTGCAGAAGGGGA		
	(R) CGTCAGTGACAGACCAGAGAGC		
	(F) CCTACGGGAGGCAGCAGTAG		
	(R) CAACAGAGCTTTACGATCCGAAA		
	(F) CGTGCTGTCTCGCCTGAAAATA		
	(R) ACTCACGATAACGCTGCAAGAC		
	(F) CCATGTGTAGCGGTGAAATGC		
	(R) TCATCGTTTACGGCGTGGAC		
	(F) CTTACCAGGTCTTGACATCCCG		
	(R) ACCCAACATCTCACGACACGAG		
	(F) AGCGTTGTCCGGATTTATTG		
	(R) CTACGCATTTCACCGCTACA		

## 4 Discussion

### 4.1 Biofilm formation

The oral cavity is a dynamic environment constantly exposed to external factors such as foods and drinks, which relies on its normal oral microbiota to maintain microbial homeostasis and overall oral health. Disruption of this balance can lead to dysbiosis of the oral microbiota and promote the formation of biofilm on dental enamel. This biofilm, known as dental plaque, provides an environment conducive to the proliferation of acidogenic and aciduric bacteria. Their metabolic interaction with fermentable carbohydrates leads to the progressive demineralization of calcified dental tissues, marking the onset and progression of dental caries, one of the most prevalent oral diseases worldwide (9, 10). Among the cariogenic bacteria, *S. mutans* is recognized as the most significant contributor to dental caries. A key virulence factor of *S. mutans* is its ability to form biofilms on tooth surfaces through the production of glucosyltransferases (GTFs) and fructosyltransferases (Ftf), which are essential for sucrose-dependent adhesion. The key GTF enzymes, encoded by *gtfB, gtfC*, and *gtfD*, synthesize extracellular glucans from fermentable carbohydrates. These glucans create specific binding sites that facilitate bacterial colonization and contribute to the formation and stability of cariogenic biofilms. These processes underscore the critical role of *S. mutans* in the development of dental caries (11, 12).

The adherence of *S. mutans* to the tooth surface involves both sucrose-dependent and sucrose-independent mechanisms. In sucrose-dependent adhesion, glucosyltransferases (Gtfs), particularly gtfB and gtfC, synthesize extracellular glucans from sucrose, forming a sticky matrix that anchors bacteria to the enamel and to each other (13, 14). These glucans also serve as a scaffold for other microbes and enhance interbacterial cohesion, enabling the maturation of a structurally stable biofilm. Moreover, these enzymes contribute to the formation of water-insoluble glucans that provide mechanical strength and acid retention capability within the plaque. The Gtfs are regulated by environmental conditions such as carbohydrate availability, pH, and stress signals. In contrast, sucrose-independent adhesion involves specific bacterial surface proteins like SpaP (also known as P1 or antigen I/II) that bind to salivary agglutinin glycoproteins (SAGs) coating the tooth surface. This interaction facilitates initial colonization even in the absence of dietary sucrose and primes the biofilm site for later glucan-mediated accumulation. Additionally, this process is aided by lipoteichoic acids (LTAs) and other surface adhesins that stabilize weak initial bonds, allowing *S. mutans* to persist during fluctuating nutrient conditions. Together, these mechanisms allow *S. mutans* to establish residence in the oral cavity under both sucrose-rich and sucrose-limited conditions, providing a dual advantage in biofilm initiation and progression (15). Sucrose-independent mechanisms support early-stage biofilm formation by enabling initial bacterial attachment via SpaP-mediated interaction with salivary glycoproteins. This establishes a foundational layer for subsequent polysaccharide-mediated aggregation in the presence of sucrose, facilitating a more robust biofilm structure (29, 30). The research by Fujiwara et al. (1996) reported that deletion of *gtfB* and *gtfC* led to a significant reduction in biofilm formation, characterized by minimal bacterial accumulation and limited polysaccharide synthesis *in vitro* (16). This finding underscores the essential role of these genes in synthesizing water-insoluble extracellular polysaccharides (EPS), which contribute to the structural integrity of cariogenic biofilms. Supporting this, inhibition of *gtfB* and *gtfC* by ActG, a protein acetyltransferase (KAT) in *Streptococcus mutans*, also resulted in markedly reduced EPS production and biofilm formation. Notably, ActG inhibition did not affect planktonic growth in either the mid-logarithmic or stationary phases, aligning with earlier findings that GTF deletion impairs biofilm development without compromising planktonic viability (16–18). Collectively, these observations suggest that targeting GTF activity is a promising strategy to inhibit biofilm development without adversely affecting the overall growth of *S. mutans* in its planktonic state (31).

Another critical component in *S. mutans* sucrose-dependent biofilm formation is the group of glucan-binding proteins (Gbps), play a key role in mediating the interaction between *S. mutans* and glucans. Gbps facilitate the binding of *S. mutans* to glucans synthesized *in situ*, thereby complementing the role of cell-associated glucosyltransferase (GTF) enzymes. This cooperative interaction between GTFs and non-GTF Gbps is essential for efficient adherence, colonization, and stabilization of the biofilm matrix, underscoring their importance in sucrose-dependent biofilm development (19, 20).

Mattos-Graner et al. (2001) demonstrated that the depletion of *gbpB* significantly disrupted the early stages of sucrose-dependent biofilm formation, impairing processes such as cell division and other physiological mechanisms critical for the transition from planktonic growth to biofilm establishment (21). These findings underscore the pivotal role of Gbps, particularly *gbpB*, in the initial organization and structural development of *S. mutans* biofilms (21, 22). Moreover, the *gbpC* gene encodes a surface-associated protein that mediates dextran-induced aggregation. The absence of *gbpC* led to reduced biofilm biomass and bacterial aggregation, indicating its role as a key receptor for glucans. This highlights the critical contribution of *gbpC* in maintaining the structural integrity and aggregation of *S. mutans* within the biofilm matrix, further reinforcing the essential roles of Gbps in biofilm formation (23, 24).

*VicK* is a histidine protein kinase in *S. mutans* that plays a pivotal role in biofilm formation by sensing and transmitting chemical signals to downstream regulatory proteins, such as *VicR* and *CovR* (25). These regulatory proteins influence the transcription of biofilm-associated genes, including *gtfB*, *gtfC*, *ftf*, and *gbpB*. Deng et al. (2021) reported that deletion of the *vicK* gene leads to a significant reduction in biofilm formation and exopolysaccharide (EPS) production, with EPS primarily accumulating around the bacterial cells (26). Additionally, the molecular weight and monosaccharide composition of EPS were markedly altered. *In vivo*, the resulting biofilms were sparse and associated with reduced dental caries severity. Most EPS synthesis-related genes showed downregulated expression following *vicK* deletion, with the exception of *gtfB*. These findings highlight the crucial role of the *vicK* gene in promoting biofilm development and caries progression, potentially through its regulatory effects on EPS metabolism, including synthesis and structural modification (27, 28).

**Figure 3:**
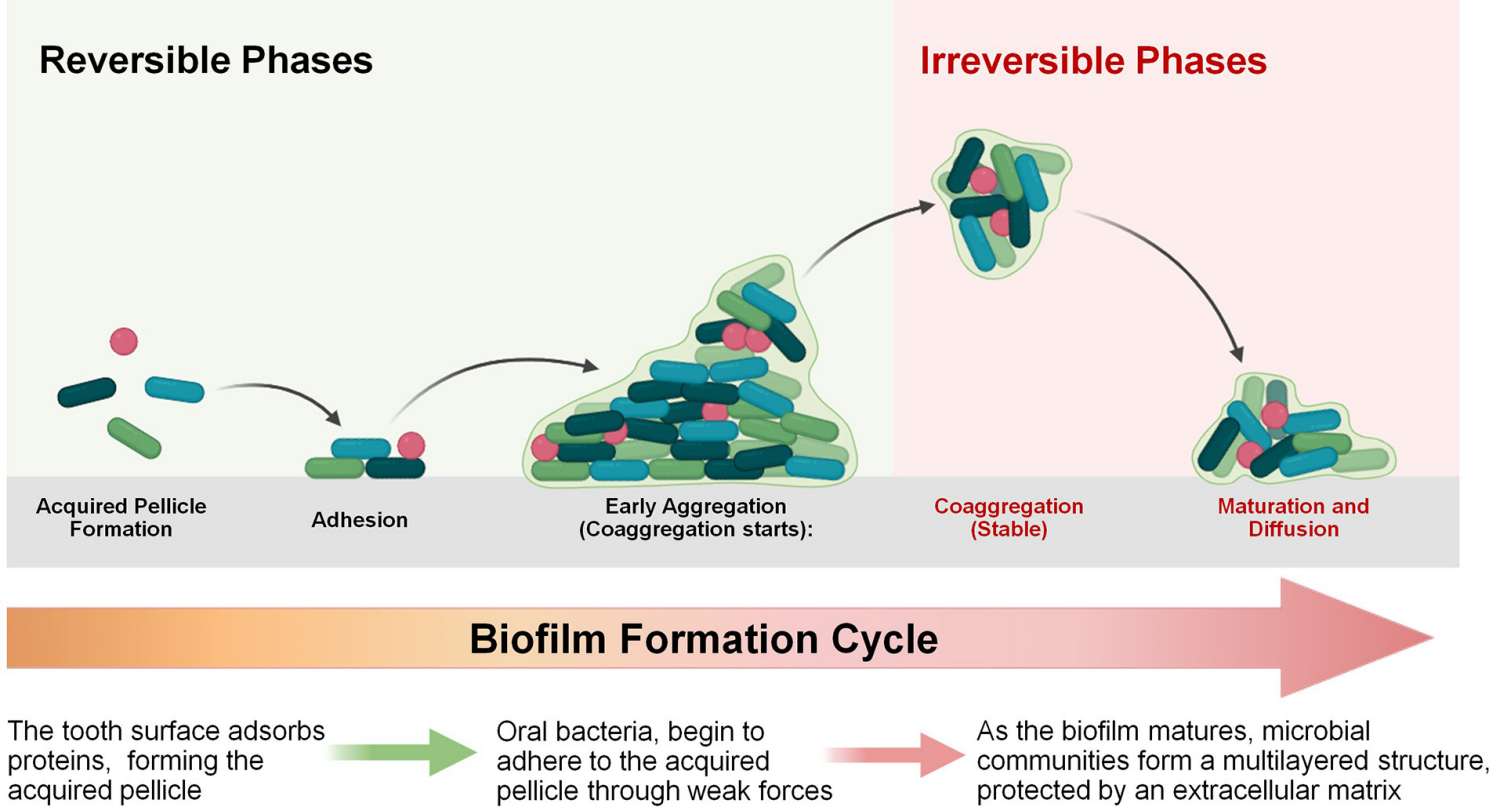
Biofilm formation cycle. The process begins with the formation of the acquired pellicle and initial bacterial adhesion, which are reversible. As the biofilm matures, bacteria coaggregate and develop a stable, multilayered structure protected by an extracellular matrix, marking the irreversible phase.

*S. mutans* has evolved multiple regulatory systems to adapt to environmental stress, among which. the *LiaSR* two-component signal transduction system plays a pivotal role, particularly in biofilm formation and stress adaptation. The *liaR* gene products are essential for cellular response to stressors, including those that damage the cell envelope (32). This system controls related pathways that enable *S. mutans* to tolerate acidic environments, resist antibiotics, and withstand exposure to detergents. Within the oral environment, *LiaSR* contributes to the growth and survival of the bacterium, providing robust defenses against acid-induced stress, antimicrobial agents, and structural damage (33, 34)*.* Additionally, the phosphotransfer from *LiaS* to *LiaR* is crucial for the activation of the *vicRKX* genes. Studies have shown that the *VicRKX* two-component signal transduction system (TCSTS) influences the *ComCDE* system and the expression of genes such as *gtfB*, *gtfC*, and *gtfD*. *comD* encodes a histidine kinase receptor that responds to competence-stimulating peptide (CSP), while *comE* encodes an intracellular response regulator that mediates the expression of related genes (32). Upon activation, *ComE* promotes the synthesis of mutacins IV and V and enhances genetic competence, thereby contributing to the pathogenicity and biofilm formation of *S. mutans* (35)*.* Suppression of these regulatory systems compromises *S. mutans*' ability to respond to environmental stress, resulting in decreased cell persistence and disrupted biofilm development. Ultimately, this impairs the bacterium's capacity to maintain its virulence and long-term survival in the oral cavity (34). Figure 3 depicts the biofilm formation cycle, transitioning from reversible to irreversible phases. In the reversible stages, bacteria first adhere to the acquired pellicle, followed by attachment and early aggregation. The irreversible phases involve stable coaggregation, where the extracellular polymeric matrix forms, leading to biofilm maturation. The cycle concludes with bacterial dispersal, enabling colonization of new surfaces. This figure highlights the key stages critical to biofilm stability and pathogenicity. The LiaSR system is enhanced by upstream activation of the VicRKX two-component system. Environmental stimuli sensed by VicK initiate phosphorylation cascades that upregulate *liaSR* expression, promoting resistance to envelope stress and supporting robust biofilm architecture (36–38).

In addition, the increasing prevalence of multidrug-resistant (MDR) strains of *S. mutans* aligns with global concerns raised by WHO regarding antimicrobial resistance (39, 40). The WHO's Global Antimicrobial Resistance Surveillance System (GLASS) emphasizes the urgency of addressing antimicrobial resistance across pathogens, including those implicated in oral infections such as dental caries (41). Biofilm-mediated resistance mechanisms in *S. mutans* further complicate treatment approaches, underscoring the importance of alternative therapeutic strategies like smart materials, and advanced computational approaches including AI-driven identification of anti-biofilm compounds (5–8, 42, 43). Such measures are important to mitigating the rise of MDR strains and enhancing oral health care globally.

### 4.2 Quorum sensing

Quorum sensing is a fundamental communication mechanism that enable bacteria to adapt to their environment by facilitating communication within a population. This process allows bacterial cells to synchronize gene expression in a cell density-dependent manner, enhancing their survival and adaptability. It operates by producing, releasing, detecting, and responding to signaling molecules known as autoinducers or self-inducers, which act similarly to hormones (44). In *S. mutans*, the quorum-sensing Com system plays a critical role in regulating biofilm formation and structural organization. This system functions most efficiently during active cell growth within the biofilm and involves the synthesis of competence-stimulating peptides (CSP), which are detected via the two-component signaling system *ComDE*. This intercellular signaling highlights the essential role of quorum sensing in the development, stability, and maintenance of *S. mutans* biofilms (45).

The ABC transporter complex, particularly ComAB, is responsible for processing the comC-encoded propeptide into a 21-amino acid competence-stimulating peptide (21-CSP), which is then cleaved by SepM into its active 18-amino acid form. This active CSP binds to the membrane-bound histidine kinase receptor ComD, initiating a phosphorylation cascade through the response regulator ComE, which in turn activates the transcription of virulence and competence genes involved in biofilm development (46). The activity of this ABC transporter system is favored during exponential bacterial growth, under nutrient-rich conditions with sufficient sucrose availability and high cell density. These environmental factors stimulate quorum sensing by enhancing CSP production and release. Additionally, acidic stress and oxidative stress conditions in mature plaque biofilms can further enhance CSP-mediated signaling, allowing *S. mutans* to coordinate biofilm-specific gene expression and genetic competence (47–50). Pourhajibagher et al. (2022) demonstrated that quorum quenching using blue laser and N-QCT suppressed the expression of QS-related genes (comA, comB, comDE), alongside the virulence gene gtfB, confirming the role of this signaling system in biofilm-associated virulence regulation (51).

### 4.3 Acidogenicity and acid tolerance

*S. mutans* is a highly acidogenic bacterium, meaning it can rapidly ferment dietary carbohydrates, particularly sucrose, glucose, and fructose into organic acids such as lactic acid (15). This acid production leads to a significant reduction in local pH, contributing to the demineralization of enamel and the initiation of dental caries. Key enzymes involved in acidogenesis include lactate dehydrogenase (ldh) and pyruvate formate lyase, which enable the bacterium to metabolize sugars under both aerobic and anaerobic conditions. Acidogenic activity is further enhanced by the presence of extracellular glucans that retain fermentable substrates within the biofilm matrix, creating a localized reservoir for sustained acid production. *S. mutans* is also aciduric, meaning it can tolerate and thrive in low-pH environments where many other oral commensals fail to survive (52). This aciduric property is critical for its dominance in cariogenic biofilms (53). The ability to maintain intracellular pH homeostasis under acidic conditions is regulated by several stress response systems, including F-ATPase proton pumps, two-component systems such as liaSR and vicRK, and membrane-associated proteins that reinforce cell envelope integrity. Additional protective systems, including the arginine deiminase pathway and malolactic fermentation, generate alkaline by-products that counteract cytoplasmic acidification. These mechanisms allow *S. mutans* to sustain intracellular pH stability, metabolic activity, and genetic competence within the acidic microenvironment of the dental plaque (54). Upregulation of acid tolerance genes is often observed under low pH, confirming their role in long-term survival. Moreover, environmental stress conditions such as acidity and nutrient limitation can further stimulate the expression of virulence genes, enhancing the pathogen's persistence and contributing to its role in dental caries development (55). Understanding these acidogenic and aciduric mechanisms is crucial for designing effective anti-caries strategies that disrupt the ecological advantages of *S. mutans* without affecting the balance of the oral microbiota (56, 57). Emerging technologies, including AI-based drug discovery, fluoride-releasing smart materials, silver diamine fluoride, and bioactive restorative agents, are being investigated to neutralize acidic microenvironments and impair biofilm persistence in caries-prone sites (5–8, 58–62).

## 5 Conclusion

This systematic review underscores the critical role of specific virulence genes in biofilm formation by *S. mutans*, a key contributor to dental caries. Genes involved in glucan synthesis (*gtfB*, *gtfC*, *gtfD*), glucan-binding (*gbpB*, *gbpC*), and regulatory systems like *vicRK* and *liaSR* are pivotal in adhesion, extracellular polysaccharide production, and environmental stress adaptation. Targeting these genes through emerging strategies, such as quorum-sensing inhibitors and anti-virulence agents, offers promising potential to reduce biofilm formation and pathogenicity. Importantly, these approaches can disrupt pathogenicity without compromising bacterial viability, thereby preserving the oral microbiome's ecological balance and providing novel pathways for therapeutic intervention in dental caries management.

Future research should focus on validating the *in vitro* findings through *in vivo* studies and clinical trials to assess the real-world effectiveness of targeting *S. mutans* virulence genes. Exploring innovative anti-virulence strategies, such as gene-editing technologies and quorum-sensing inhibitors, is essential to evaluate their safety and long-term impact on biofilm formation. Additionally, integrating omics approaches, including transcriptomics and proteomics, will provide a deeper understanding of the mechanisms driving *S. mutans* pathogenicity and biofilm formation. Collaborative studies combining these strategies with conventional treatments could improve therapeutic outcomes while preserving the oral microbiome's ecological balance. Furthermore, addressing potential resistance to these therapies will be key to ensuring their future success in preventing dental caries.

### 5.1 Limitations of the study

Most included studies focused narrowly on specific virulence genes or isolated pathways, limiting comprehensive insights into broader gene networks involved in *S. mutans* biofilm formation (19). Additionally, variability in experimental conditions (such as culture media, sucrose concentration, methods for gene expression analysis, and duration of biofilm maturation) introduced heterogeneity, potentially affecting the consistency and applicability of results. Another limitation highlighted by QUIN assessment was the absence of standardized sample size calculations and operator information, potentially affecting reproducibility and generalizability of findings (63). Furthermore, all studies included were *in vitro*, which restricts direct applicability to clinical practice. Hence, validation through clinical trials and *in vivo* studies is necessary for more robust and applicable conclusions.

## Data Availability

The original contributions presented in the study are included in the article. Further inquiries can be directed to the corresponding author.
